# Necessity as the Catalyst of Change: Exploring Client and Provider Perspectives of Accelerated Implementation of Telehealth by a Regional Australian Community Service Organisation during COVID-19 Restrictions

**DOI:** 10.3390/ijerph182111433

**Published:** 2021-10-30

**Authors:** Leah Ayres, Lindi Pelkowitz, Perlin Simon, Sandra C. Thompson

**Affiliations:** 1 Western Australian Centre for Rural Health, University of Western Australia, Geraldton, WA 6530, Australia; lindi.pelkowitz@uwa.edu.au (L.P.); sandra.thompson@uwa.edu.au (S.C.T.); 2 Centacare Family Services, Geraldton, WA 6530, Australia; ceo@centacaregeraldton.org.au

**Keywords:** community services, welfare, telepractice, Australia, COVID-19 pandemic, regional, service experience

## Abstract

Community services have played a significant role in supporting the psychosocial health and well-being of vulnerable populations during the SARS-CoV-2 (COVID-19) pandemic. To meet increased community needs, organisations were required to rapidly modify service provision, often using remote delivery systems. This in-depth study, undertaken early in the pandemic, explored staff and clients’ experiences of adapting to using telehealth to provide and access services in one regional social services agency. Semi-structured interviews from 15 staff and 11 clients from a regional not-for-profit agency in Western Australia were recorded and transcribed. Inductive coding, and thematic analysis identified eight subthemes, with experiences and perceptions of telehealth varying substantially among staff and client groups. Distinct benefits and challenges were associated with telehealth. Participants highlighted tensions and complexities and commented on the place of telehealth in the community service sector. Clients expressed the importance of relationships and communication. This study provides in-depth insights into the contextualised experiences of staff and clients during a time at which change was both enabled and necessary. The findings highlight the need for tailored service delivery; choice; client collaboration; ongoing staff training relating to telehealth; and guidelines specific to telehealth in the community service sector.

## 1. Introduction

The COVID-19 pandemic has drastically affected individuals, families and communities worldwide [1,2]. Within Australia, community service organisations, alongside government initiatives, have played a notable role in responding to the social impacts of the pandemic such as increased unemployment, mental health issues and social isolation [3,4]. While working to meet increasing community needs, organisations were also required to rapidly restructure service arrangements in accordance with social distancing guidelines [3,5]. This led many to implement telehealth as a means of ongoing service delivery [3].

Telehealth is defined as the “delivery of health care services, where patients and providers are separated by distance” [6] (para. 1). Wade [7] distinguishes between two forms of telehealth: asynchronous and synchronous; the former involves no ‘real-time’ interaction between provider and client (e.g., emails), while the latter is characterised by simultaneous interactions (e.g., telephone and videoconferencing). Although telehealth is commonly conceptualised in a medical context, its meaning has extended over time to include broader understandings of health and well-being [5]. Alternative phrases have emerged with this expansion; telepractice is regarded as more general term that encompasses the use of information and communications technology [ICT] to deliver services across a range of fields [8]. In this study, the terms telehealth and telepractice are used interchangeably to mean the use of synchronous communications, particularly teleconferencing and telephone, to deliver community services. Community services can be broadly defined as agencies that assist people who may be experiencing vulnerability by supporting their well-being and relationships, access to resources, and social participation [9].

Telepractice has been widely studied across multiple disciplines; over the decades, substantial literature has emerged commenting on its acceptability and efficacy [8,10]. A recent review of telehealth literature from the last decade found that although some studies reported telepractice outcomes to be equally, if not more, effective, the “strength of this evidence is unclear” [8] (p. 4). Even so, it is widely agreed that telehealth is a promising mode of service delivery with distinct advantages [7,8,11,12,13]. Commonly reported benefits for service users include improved access to services; enhanced quality, frequency or timeliness of services; reduced costs; and increased convenience [8,11,12,13]. Telepractice has also been found to benefit practitioners, allowing greater “access to continuing education and professional development activities, [and] the ability to provide an enhanced local service” [12] (p. 277). Along with advantages, the barriers and challenges associated with telehealth have been described within the literature. These have notably included clinician and provider preferences for in-person services, limited resources and funding, insufficient infrastructure, lack of reliable connection or broadband, and ethical and legal concerns (e.g., data security and privacy) [8,11].

Within the Australian context, research into telehealth holds particular importance for those residing in rural areas [11]. Existing studies have established that rural, regional and remote populations can benefit from using telepractice to access a range of medical and allied health services [12]. However, there are also distinct challenges for those living rurally, with Thomas et al. [14] (p. 6, 8) reporting a considerable “Capital-Country Gap” in digital inclusion: “whether a person can Access, Afford and have the Digital Ability to connect and use online technologies effectively”. Considering this subset of the population stands the most to gain from telepractice [15], ongoing research into the perspectives of stakeholders in regional areas is crucial.

Despite the extensive literature published about telehealth, its actual implementation was still in its “infancy stage” prior to COVID-19 [16] (p. 8). The pandemic and subsequent restrictions catalysed digitalisation and adaptation across numerous sectors, with telehealth operating at a “much larger scale than before” [17] (p. 9). This markedly impacted the Australian community services landscape, with 96% of services shifting some aspect of their service delivery from face-to-face practice to other modes such as telepractice [3]. According to the recent Australian Community Sector Survey, almost a quarter of services “reported that their entire service shifted from face-to-face” [3] (p. 9).

This rapid transition was commented on by Sanders [5], who reported on the experiences of human service workers in regional Victoria; practitioners expressed both positive and negative aspects of shifting to telepractice during the pandemic. The workers commented on the benefits of increased accessibility, improved communication between agencies and professionals, and a greater work/life balance when using flexible modes of service delivery [5]. However, practitioners also had concerns about ICT issues, communication with service users, and the overall quality of services [5]. While Sanders [5] offers insight into the experiences of practitioners, the paper does not provide information about the service areas under comment. As the human service sector is diverse in terms of practice and client needs, research which explores the contextualised experiences of service workers could contribute to the ongoing discussion of telepractice in this field.

In addition, there is a need for further qualitative research into clients’ experiences of accessing community services through telepractice. Callis et al.’s. [1] (p. 20) recent 100 Families WA COVID-19 report found “there was substantial variation in people’s experiences of services during COVID-19”. Respondents’ perceptions of changes to community services were divided relatively evenly between positive and negative; 46% of families found the shifts either more or wholly positive, while 54% indicated that they were more or wholly negative [1]. This is noteworthy as other fields and disciplines, such as medicine, have noted that service users, alongside providers, have widely embraced telehealth [18,19]. An in-depth exploration of individuals’ experiences of using telepractice to access community services could provide insight into this disparity and the unique nature of telehealth in this context.

To date, most of the research into telehealth has focused on medical, psychiatric, nursing, and allied health applications. Few studies have examined the use of telehealth by community service organisations, especially within the regional Australian context. Centacare Family Services—Geraldton [CFS], the setting for the current study, was uniquely positioned as a regional organisation which offers multiple community services including counselling, family and parenting support, family separation services, and emergency relief [20].

CFS is a Western Australian [WA] not-for-profit agency that services a large catchment area across the Midwest and Gascoyne regions. On the 16 March 2020, it became one of the first organisations in Geraldton to transition almost entirely to telepractice. While CFS, like many regional agencies, had some elements of telehealth established prior to COVID-19, this was limited in scope. Telephone was mainly used to work with clients residing outside of Greater Geraldton or the Midwest region; apart from this, policies, practices, and infrastructure related to telehealth were minimal. As such, many staff and clients had little prior experience using telepractice to deliver and access community services.

Although the direct impacts of COVID-19 and the subsequent restrictions were relatively short-lived in WA, the service landscape has been markedly affected. Thus, an examination of the use and responses to telehealth within this context can add to the growing evidence base surrounding the effectiveness, complexities, and implications of telepractice. Considering this, the aim of this study was to investigate the service response to COVID-19, and how it impacted staff and clients’ experiences of providing and accessing regional community services. As telepractice emerged as a key means of ongoing service delivery, this paper captures the response to two core objectives: (i) to explore clients’ experiences of using telehealth to access community services; and (ii) to understand staff’s experiences of delivering community services via telehealth. The findings indicate the need for more tailored service delivery, specified training for staff, and organisational mechanisms for client collaboration and choice.

## 2. Methods

### 2.1. Research Design and Methodology

This study used a qualitative approach with insights from an interpretivist paradigm, which recognises the “importance of people’s experiences, meanings, understandings and interpretation of events” [21] (p. 84). This approach suited the research which aimed to examine participants’ experiences and perceptions in relation to the uptake and use of telehealth for community service provision. In-depth interviewing—a method which aligns closely with interpretivism [22]—was conducted to elicit thick descriptions and allow participants to articulate their experiences in their own words. Data were then inductively coded and thematically analysed, drawing on Brooks and King’s [23] template style of analysis. Basic statistical analysis was also used to capture participants’ demographic details.

### 2.2. Sampling and Participants

Participants were sourced using purposive maximum variation sampling, which sought to include service providers and clients from different service areas: counselling, family support, family separation, and emergency relief. This sampling framework, a “more flexible variant of stratified purposive sampling” [24] (p. 93), was suited to the research as it gathered experiences from multiple subgroups and facilitated comparative analysis. Recruitment of service providers involved sending an email invitation which included a plain language description of this study and copies of the participant information and consent forms.

Clients who met the inclusion criterion were informed of the research by the organisation’s clinical and administrative staff. The inclusion criterion was any client aged ≥18 years who engaged with the organisation between March and May 2020; this was deliberately broad to elicit a wide range of client voices. With their consent, staff provided interested clients’ contact details to L.A. We then called or emailed these clients to discuss this study further and provide copies of the participant information and consent forms.

### 2.3. Data Collection

Separate interview guides were constructed for the client and service provider subgroups, ensuring questions were relevant and appropriate for each. Interviews with service providers focused on their perceptions of how clinical practice and service delivery changed, any related benefits or challenges, client reactions, and their experience of the organisation’s response. Interviews with clients centred around their experiences of accessing telehealth services, anything helpful or challenging about the changes to service delivery, and what they would like to see in relation to future service provision.

Overall, 26 semi-structured in-depth interviews were conducted by L.A. over a five-month period to elicit detailed accounts of each participant’s experience. Interviews were scheduled at the convenience of participants and were conducted by telephone, videoconferencing, or in-person, generally lasting approximately 30 min. All interviews were audio recorded for accuracy and transcribed verbatim; verbal consent was given prior to data collection and captured on tape. Participants were offered the opportunity to review their transcripts; two participants did so, with one providing comments and revisions.

Demographic details (age, gender, cultural identity, and employment) were sought from participants. While the original intent had been to include this information, it was felt that certain details such as cultural identity would make participants and especially participating service providers readily identifiable within the organisation. Hence, to protect the privacy of participants, identifying information was omitted or changed in reporting and pseudonyms used to ensure client and service provider anonymity.

The duration of data collection was guided by inductive thematic saturation—a form of saturation that requires ongoing critical reflection on the generation of new codes and themes from the data [25]. When used in this way, saturation is conceptualised as an incremental process rather than a fixed point [25]. We considered data collection sufficient when a rich network of themes was generated which identified commonalities in meanings and experiences, as well as complexities and differences depending on participants’ service area. This use of saturation was appropriate to the study design as it aligns with its inductive methods [25].

### 2.4. Data Analysis

Inductive coding and thematic analysis (TA) were used to manually organise, interpret, and generate themes from the data. This method was selected for its flexibility and alignment with this study’s aims; Nowell et al. [26] note TA is especially useful for examining participants’ perceptions and exploring the commonalities and differences across data. This fitted well with our research objectives and focus on multiple subgroups’ experiences. We were guided by Template Analysis—a ‘codebook’ style of TA which offers a systematic yet flexible approach within a qualitative paradigm [27,28].

As per Brooks and King’s [23] procedure for Template Analysis, our process began with data familiarisation. This involved reading and re-reading transcripts as well as re-listening to segments of the audio-recordings [29]. An inductive method was then used to code the data section by section, with initial codes ranging from a few words to several sentences in length. Coding techniques included manually underlining segments or ideas that were repeated or emphasised by the participant and noting codes in the margins.

After preliminarily coding all transcripts, codes were compared and further developed to produce an initial template; this included broad overarching themes and successive themes, which were narrower and more focused. The refinement of the template occurred iteratively as it was applied to the data and modified accordingly. This involved reorganising existing themes, generating new themes, and removing those that lacked relevance. The template was reviewed and discussed by L.A., L.P., and S.T. at each stage of refinement to facilitate critical exploration of the themes generated and discussion of alternate interpretations. A final template focusing on participants’ experiences of telehealth service delivery was agreed upon and applied to the data set. This served as the basis for analysis and was supported by excepts from the data. The quotations were identified and selected based on being richly detailed or elucidating the meaning or context of the themes. Basic descriptive statistical analysis—mean and standard deviation—were used to provide an overview of participant characteristics.

### 2.5. Methodological Rigour

Several strategies were employed to promote rigour; ongoing peer debriefings were utilised at all stages of the research to facilitate reflection on researcher subjectivity and bias. Thick and contextualised descriptions of participant’s perceptions and experiences were also sought, ensuring sufficient depth was reached for analysis, promoting the credibility of the research [30]. Validity was also promoted through data triangulation [31]; multiple perspectives were gained from service providers and clients from different service areas which allowed for a more comprehensive understanding of experiences. Analysis was refined collaboratively through team meetings to further increase the soundness of the interpretive approach.

### 2.6. Ethical Considerations

The research project was approved by the University of Western Australian Human Research Ethics Committee, project number RA/4/20/6186. The voluntary nature of this study was made explicit to all participants. Both service providers and clients were assured that their decision to participate or decline participation would not result in any negative consequences such as decrease in support or disadvantage in employment. While the director of the organisation was an investigator for this study, she was appropriately distanced from data collection and did not have access to staff or client interviews or transcripts.

## 3. Results

### 3.1. Participant Characterisics

Of the 29 staff at CFS, 15 (51.7%) expressed interest in this study, and all participated. The majority (93%) were female, and providers had an average age of 48 years (mean = 48.1, standard deviation (SD) = 15.1). Staff were from multiple service areas: counselling (*n* = 6), family and parenting support (*n* = 1), family separation services (*n* = 5), and emergency relief [ER] (*n* = 1). Two worked across more than one service area. At the time of interview, nine staff were working as practitioners delivering services directly to clients, three were in support roles that involved client contact, and three were not in client-facing roles. The length staff had been employed at CFS ranged from less than a year to over 25 years.

Fifteen clients expressed interest in this study, with 11 consenting to be interviewed. Again, most were female (82%), with an average age of 41 years (mean = 41.2, SD = 11.9). Two clients lived outside of the Midwest region; both were accessing family separation services and had ex-partners living in Greater Geraldton. One client did not access services directly but reported on the experiences of her children. Clients utilised a range of services: counselling (*n* = 5), family and parenting support (*n* = 3), and family separation services (*n* = 3). The participant characteristics for both client and staff samples are summarised in Table 1.

### 3.2. Staff Experiences of Using Telepractice to Deliver Community Services

Four main themes were generated from staff experiences of delivering community services by telehealth: (1) benefits and opportunities arising from telepractice; (2) challenges and barriers encountered by providers; (3) staff attitudes and the perceived value of face-to-face practice; and (4) complexities and tensions within the community services context.

#### 3.2.1. Benefits and Opportunities Arising from Telepractice

- Increased Accessibility for Clients

Staff of most service areas discussed the value of telepractice in increasing the accessibility of community services for clients, especially those living outside town. This finding is consistent with previous research which outlines the benefits of telehealth for rural and remote Australians [12]. Several staff spoke highly of telehealth’s potential to facilitate greater support for individuals and families residing on farms or in remote communities, and its usefulness even beyond the context of COVID-19.

*There are people out in Meekatharra and on stations that are a thousand kilometres out who can’t possibly get in for counselling. […] So now that we’ve got this system working so well, I can see us offering this to those external communities (S03).*

This issue of accessibility cannot be understated within the Australian context, with WA having one of the lowest population densities globally [32]. Locality can profoundly impact an individual’s ability to engage with services, and staff identified long travel distances and insufficient transport as barriers for rural clients. As one provider succinctly put it: *“it might be telephone or nothing” (S10).*

Staff believed other client groups, such as those with chronic conditions, mobility issues, or mental health conditions also benefitted from increased accessibility of services.

*…for a client who might not have enough energy to get up, get dressed and go to their appointment—now they can do it from their lounge, it’s easier for them (S04).*

Telehealth was also useful for families facing structural barriers such as lack of private transport *“there are clients that don’t have their own transport, who do rely on public transport […] so, you know, getting to an appointment is harder for some people” (S02)*.

Those clients accessing emergency food relief and financial support services were identified as particularly benefiting from this mode of practice. Others benefitting were clients who otherwise struggled to access services due to their schedules, including shift workers and parents, with some requesting to continue telehealth beyond the mandatory lockdown period.

- Increased Options for Practice

Providers identified ways to extend and enhance service delivery through telehealth which was seen as an additional ‘tool’ which could be integrated into practice, providing both clients and practitioners with more options. One staff member involved in a parenting information and support group explained how telehealth could be used to connect with a greater range of speakers and experts *“[…] we realised how easy Zoom was and that we can utilise guest speakers from interstate or in the city” (S05).*

Given the limited number of services and professionals within regional and rural contexts, this was considered invaluable. The ability to connect with professionals, who would otherwise be inaccessible, was viewed as an opportunity to provide clients with both a broader range of information and specialised expertise.

Staff involved in family dispute resolution [FDR]—a mediation service for separating families—found telepractice especially advantageous. One topic repeatedly raised was the way telehealth could be implemented to increase safety for participants and negotiate power disparities between those mediating.

*…running mediations by Zoom can be quite good when there is a risk of the parties becoming heightened or you know, one party’s perhaps going to dominate or talk over the other because you can mute them immediately […] you can turn off their videos if they start to become threatening or they look like they’re intimidating the other party […]. You can do that with a click of the button so—as a facilitator—that’s a handy tool to have (S01).*

Telehealth also offered FDR practitioners an alternative to traditional ‘shuttle’ mediation, a form of FDR where clients deemed unsuitable for face-to-face services are in separate rooms with the facilitator ‘shuttling’ between them. Telepractice was seen as an opportunity to increase the safety and effectiveness of mediation in cases of high conflict or risk.

*I found that [telepractice] is quite a good platform for some mediation, where it’s not quite suitable for face-to-face […] but better than doing shuttle. So I think it opened a lot of new opportunities to think that some mediations could be done by Zoom as a safety factor or a different opportunity rather than just face-to-face or shuttle (S08).*

Providers delivering FDR identified distinct benefits of videoconferencing over phone calls reporting it allowed them to gain a better sense of clients’ comfort levels and work more collaboratively using online functions such as screen sharing or writing on a digital whiteboard.

*You can pick up on a lot of that body language […] you can sort of see if someone’s getting agitated, if someone’s not coping very well, you know. There’s a lot of signals there. […] we can share the whiteboard or the screen we’re working on. And that makes a huge difference because the people can actually see what we’re writing, they get to read it. Whereas on the phone, it’s just verbal, and I think that makes it really quite more difficult (S08).*

These features were used to enhance the service for clients who lived rurally or outside the Midwest. Where staff had only used phone in the past, use of videoconferencing during COVID-19 highlighted the potential for improving and extending service delivery by embracing new platforms.

#### 3.2.2. Challenges and Barriers Encountered by Providers

- Technical barriers and disparities

ICT issues were a significant barrier for some providers, impacting on the quality of their service and, in some cases, inhibiting them from working with clients altogether.

*[…] the biggest issue was poor internet. So that would sabotage the session […] the picture would freeze, the picture would fade, they’d drop out, I’d miss some of the audio […] I think 50% of my cases that I did visual audio had some sort of interference (S09).*

This echoes findings from existing research, with technological issues as a barrier to the implementation and ongoing use of telepractice [33,34]. However, this was not the case for all, with some reporting little to no technical issues “*ninety-nine times out of one hundred it worked perfectly well. Just straight on to the client, the client can see you easily… we’re both side-by-side on the screen” (S03).*

This disparity may reflect clients’ broadband access, with providers explaining there was little they could do when clients had poor connection or reception *“We could provide the service but if they [clients] had internet issues, if they had phone line issues, that was really out of our control” (S07).* Reliable internet is a known key determinant for access to telehealth services [16]. Thus, while telepractice can increase accessibility for some, others may be excluded from this mode of service delivery.

- Clients’ Digital Abilities and Hesitancy towards Telepractice

Another challenge was clients’ competence and confidence with using technology. Practitioners explained that digital ability, the skills required to effectively navigate and use technology, varied considerably among clients *“[…] some people [clients] indicated quite clearly some distress about being competent to use the technology. Then you had others where it wasn’t an issue, it was seamless” (S15).*

Providers felt the transition to telehealth was most challenging for clients who had high levels of vulnerability or difficulties with ICT. *“I know for some clients though who were really vulnerable it was really challenging, particularly if they had issues with technology or things, even just setting up the phone sessions could be a challenge […]” (S07).* This aligns with existing literature which asserts basic ICT skills and literacy are requisites for people to make use of digital technologies of any kind, including telehealth [14]. To navigate these challenges, some providers offered practice sessions with the aim of increasing clients’ confidence and skills with relevant technologies.

*[…] we talked them through how easy it was, you download the app, it’s free, we can do dummy runs with you beforehand so you’re comfortable with it when you get to the appointment stage (S13).*

Some staff identified clients’ attitudes towards telepractice as another challenge. Providers reported that some clients expressed hesitation or resistance to engaging in telehealth. This occurred predominately for those involved in counselling services; telepractice was considered a major obstacle and distressing for some clients. As a result, some counsellors noted decreases in their caseloads.

*I would say, out of all the clients I had, probably less than half chose to continue with online or telephone. Most people just chose not to engage during that time (S10).*

Counselling staff also reported higher rates of cancellation during this period. As one provider commented:

*…there was a definite drop in clients […] we found that a lot of people would actually rather cancel their appointments then do it by phone. Actually, I can remember a comment made by one gentleman who said, ‘it is hard enough opening up to the counsellor sitting across from them, I can’t imagine how I would open up on the phone, not seeing their face and everything’ […] I think that really stuck with me because I think that’s how a lot of people felt (S11).*

While such experiences were common for counselling staff, other service areas found clients relatively accepting of telehealth. In the area of emergency relief and food assistance, replacing in-person appointments with telepractice was considered a relief for some clients. *“They [emergency relief clients] are just more comfortable, they’re able to tell you maybe a little bit more than what they might tell you if they’re in the office” (S02).*

Similarly, clients engaging in a support group for new parents were described as enthusiastic for the online service. *“I contacted them and asked them their thoughts on ‘how do you think the group would be if we did it* via *Zoom?’ They all responded ‘yep, do it, do it, do it’. […] So, I put it out there […] and we were inundated” (S05).*

This highlights that clients held widely varying attitudes towards telehealth which impacted their engagement with services. Client apprehension was mainly identified by those within mental health services and was a significant challenge for staff.

#### 3.2.3. Staff Attitudes and the Perceived Value of Face-to-Face Services

While most staff recognised both the challenges and benefits of telehealth, attitudes and perceptions varied. Those who were less accepting of the changes focused more on limitations and viewed telehealth as an interim substitute or a substandard mode of practice.

*I believe that there is always going to be a place for face-to-face—that it will always take precedence […]. When there’s a choice, face-to-face is going to be the number one way to connect with clients. (S09).*

Conversely, those more accepting of telehealth spent time discussing the benefits and possibilities for practice.

*I’d like to continue on with an embrace of technology rather than a fear of it […] it gives the clients more options. It gives the practitioner more options […] we’re more aligned with contemporary practice (S15).*

Many factors shaped providers’ attitudes: familiarity with telepractice and technology, perceptions of efficacy, and personal characteristics. For some, prior telehealth experience appeared to influence levels of comfort and acceptance. Those familiar with telepractice described ‘slipping’ back into it with less difficulty. Some suggested that working regionally and servicing a large catchment area supported their uptake of telehealth *“It was an easy transition because, being a regional service, we have a lot of phone clients anyway […] so it was a skill I already had” (S12).*

However, this comfort level did not always translate to other modes of telehealth; some practitioners, despite extensive phone experience expressed apprehension towards videoconferencing, a less familiar platform. Formal training was identified as important to improving providers’ comfort with telehealth. One staff member explained the limitations arising from the sudden transition. *“I received absolutely no training in it […] it’s self-learning what we did, and yeah, I think that [training] could be a help” (S09).*

Providers’ uptake of telehealth was also impacted by their perceptions of its efficacy and usefulness, with those who saw benefits for clients discussing its continuation beyond COVID-19. They suggested ways telehealth could be used to improve processes and identified changes they would like to see implemented on a more permanent basis.

*[…] we were able to do phone registrations at that time and I really think it was a bit better for some of our clients […] so I think the phone regos are definitely something I’d like to see as an option for people (S08).*

Equally, where telehealth was considered less effective for clients, it influenced the provider’s attitude, leading them to express greater ambivalence and reluctance.

*I can’t really think of anything beneficial, you know, to come out of it […] I think we need the body language to see what kind of situation and state and…yeah. It was just not really working (S11).*

Moreover, personal characteristics such as openness to change appeared to inform some providers’ attitudes. Some staff indicated being more comfortable with established modes of practice. *“I was so excited to just go back to the way things were, but that’s me. That’s my personality—I’m a creature of habit, not being as open to adjustment” (S10).* Others seemed excited by the prospect of change *“we need to make certain that we’re maintaining technology […] and we’re willing to be experimental” (S15).*

Although staff attitudes varied, a common thread was the perceived value of face-to-face practice. Even those who embraced telehealth discussed the importance of working in person with clients, citing it as an intrinsic aspect of community service provision *“there’s nothing like a face-to-face interaction […] you know just tracking all those thoughts and emotions and body language that you get from a client, it’s just invaluable” (S15).*

The importance of non-verbal cues was recurrently mentioned by providers who noted that face-to-face contact allowed them to better connect with clients and ‘read’ their emotions. Additionally, the importance of the practitioner–client relationship was reiterated.

*My main focus with clients is always the relationship and making sure the client feels comfortable and is in a position to trust what’s going on—that’s most important. That could work better face-to-face than remote. I think it’ll take a lot more before the client can really allow themselves to become more vulnerable (S09).*

The relationship between providers and clients holds particular importance in the community services sector, often forming the basis for service provision. As such, establishing trust and rapport with clients is vital. Staff’s comments around this aligned with recent dialogue in the field which has considered the “intangible benefits of in-person, human contact” and the importance of maintaining quality relationships [35] (p. 403).

#### 3.2.4. The Complexities and Tensions within the Community Services Context

In line with existing literature, staff discussed additional complexities and tensions that arose when delivering services remotely through telehealth. A recurring concern was the privacy of clients and their ability to maintain confidentiality when discussing sensitive matters. This was emphasised by counselling staff.

*The biggest issue with people doing counselling at home is I can’t see who is in the room […] I rely on my client to tell me they’re in a safe, private environment, but I can’t know for sure. That’s probably the worst—my biggest concern (S10).*

Providers also discussed the added layer of complexity when working with clients who were particularly vulnerable, such as children. Practitioners described needing to have a heightened awareness of who else may be present during appointments.

*In terms of confidentiality, the other big issue was around kids. So, a lot of practitioners, I guess, just made sure they were aware that kids might have parents or carers listening in and really changed the way that they did their sessions (S07).*

In addition to privacy, staff mentioned navigating issues surrounding client safety. As experiences of family and domestic violence [FDV] are not uncommon for clients accessing community services, providers needed to ensure telehealth would be safe for victim-survivors who were residing with perpetrators. *“I was dealing with one—more than one client that was experiencing high family violence and I did feel it was especially pertinent to be more mindful of the risk people experiencing family violence would be at…” (S12).*

To manage this risk, some necessary precautions were outlined: not messaging, calling or emailing the client without explicit permission; and only contacting the client when they were already in a position of safety (e.g., in the presence of a specialist service provider or other professional). However, in some cases, telehealth was not suitable for people experiencing FDV or abuse. When discerning the appropriateness of telehealth, providers prioritised clients’ safety and well-being, taking into account the person’s circumstances.

*Obviously one of the big vulnerabilities and issues we were really concerned about was clients in DV situations, clients who, you know, we might have known had a partner in the home who was abusive or things like that, and not being able to have those phone sessions or things. I guess one way we sort of navigated that was by having the option to come in still […] each practitioner just did the best that they could and offered what they could (S07).*

### 3.3. Client Experiences of Using Telepractice to Access Community Services

Four central themes were generated from client interviews to capture the experiences of individuals from different service areas: (1) accessibility of telepractice; (2) suitability of telepractice; (3) communication and connection; and (4) client perceptions and attitudes towards telepractice.

#### 3.3.1. Accessibility of Telepractice

Notions of accessibility were recurrently discussed by clients who overall found telehealth a relatively accessible mode of practice. While staff identified technological challenges such as unstable internet as an impediment, all except one client described the technical aspects of accessing telehealth to be acceptable; in that case, the client described “*a few hiccups with the initial phone calls” (C10).* Overall, service users suggested that telepractice was easy to use “*It was simple. I just called prior to my appointment, made my payment. They would check me in, I think, and then [the provider] would give me a call in a few minutes” (C07).*

Despite this, some reflected on the barriers that exist for others in their community. *“[…] the technology’s not going to work for lower socio-economic groups either. Internet connection and, I mean, I…a lot of people still don’t have a webcam you know” (C08).* That said, some clients argued telehealth can increase accessibility for vulnerable individuals and families. The clients of a parenting support group in particular considered telepractice to be beneficial for geographically isolated women and suggested that an online platform may be more inclusive of those living rurally *“especially for the poor ladies that are isolated and out of town. […] All the ones stuck on farms and like, north or Northampton that might not have access to stuff like this” (C01).* These clients also discussed use of telepractice beyond COVID-19 to increase the accessibility of information for new parents. Concrete examples were given, such as recording guest speakers during sessions and uploading content to social media for a greater audience to access.

#### 3.3.2. Suitability of Telepractice

While most clients reported being able to access telehealth, there was a range of responses regarding its suitability. Some felt telepractice was an appropriate fit, while others found that it was incongruous with their needs or personal circumstances. For those engaged with the parenting support group, the flexibility and informality of telehealth were positives. Clients of this service, who were all mothers with young children, articulated this.

*[…] it was quite nice, you know, like not having to dress up to go out of the house. You could go off and get yourself a cup of tea in your own home…so it was a lot easier to manage (C02).*

Despite this benefit, some clients made the distinction between the online and in-person support groups *“I think the real-life mother’s group is for mum and baby, whereas the virtual one is very much for mum” (C03).*

Speaking to this difference, one client explained that while online groups offer women a platform for connecting and seeking information, they are incapable of providing the same level of socialisation for infants.

*[…] my daughter, she’s a very sort of…high needs baby and likes a lot of stimulation […] So I’d preferentially sort of go for a walk or something with her. So even though I wanted to go, you know, to hear the speaker or to see the person, it didn’t really work for me (C03).*

Similarly, issues were identified regarding the suitability of online counselling for children. In one case, despite having the ability to engage in telepractice, the family decided to suspend the children’s sessions *“We ended up stopping the counselling. […] Mainly because I knew the girls wouldn’t sit still long enough to look at the screen or talk on the phone” (C09).*

Other issues raised by counselling clients were having a suitable environment for sessions and privacy. Like staff, some clients felt they did not have an appropriate space to discuss sensitive matters that would be free of disruptions.

*Even though I…I sort of said to my family, ‘Hey I’m, you know, talking to my counsellor at this time,’ there were interruptions […] So yeah, that was a downside of it as well: unforeseen interruptions” (C10).*

Some clients also felt telehealth was incompatible with their individual needs. It was suggested that the personal circumstances that brought some clients to the service in turn shaped their experiences of telepractice. One client emphasised this when discussing their preference for face-to-face counselling.

*I felt like [phone counselling] still helped me, but I think the face-to-face helped me more. Yeah, purely because I don’t see a lot of people, yeah, so maybe if I had more family and more support around me, it might not have been that bad to have a phone call, but because that’s life for me […] I guess I kind of look forward to seeing the counsellor to get things off my chest in person (C04).*

Another client felt telehealth was unsuitable due to their experience of anxiety and trauma, which meant they felt distressed by phone calls *“I hate phones…absolutely hate them […] I start getting very panicky. I suffer from panic attacks and anxiety and with the phone I get very very very stressed out” (C05).*

#### 3.3.3. Communication and Connection

Most clients discussed the impact telehealth had on their ability to connect with practitioners or, in the case of group programs, other service users. Despite recognising the benefits, many clients felt more comfortable with face-to-face interactions. There was a perception among clients that telepractice was less personal and some found it difficult to communicate over phone or videoconference.

*[…] not having the face-to-face really was the most difficult part […] you lose that sort of emotion side of it and the genuine conversation with somebody and the focus. That’s the sort of thing that means it’s sort of harder for me to open up (C11).*

This concept of ‘opening up’ was repeatedly mentioned, with clients suggesting that they were more hesitant to discuss their personal matters when accessing services via telehealth. *“[…] it’s a bit harder to open up […] maybe you might not let everything out that’s bothering you as much” (C04).*

These experiences were expressed most by those accessing individual counselling or other forms of emotional support. For these clients, non-verbal cues were considered essential for effective communication. Many found conversing over the phone to be challenging.

*I felt it was more difficult to convey exactly how I was feeling about things, whereas when you’re face-to-face with someone you can read their emotions and yeah, it felt like I wasn’t totally getting across what I was trying to say (C10).*

Clients also reported that the lack of body language and facial expressions resulted in feelings of isolation and disconnect *“You can’t—I guess you can’t sort of see the expression on their face…Yeah. It’s just…it just seems isolated” (C04).* Yet despite the value placed on visual cues, many clients chose phone instead of videoconference—some for practical reasons but others perceived phone conversations as more casual and informal, *“the phone felt more like … more of a normal conversation…whereas video felt like you were, I don’t know, like it was an interview” (C10).*

Having an established bond facilitated effective communication even in the absence of non-verbal cues:

*I guess because I had been seeing my counsellor for so long, they know me quite well and what my voice sounds like and the way my head works [laughing] basically…so I still felt like, because we had such a long relationship prior to that, going on the phone was fine because I was sure they’d be able to read my voice and what I was saying anyway” (C07).*

However, others found communicating by phone challenging, even when they had an existing rapport with providers *“[…] to me it’s no reflection on my counsellor because I reckon I’ve got the best counsellor in—if not the world, it’d be Australia […] It just keeps going back to I don’t like phones” (C05).* The lack of body language and physical presence resulted in feelings of anxiety with clients describing feeling more relaxed and at ease when speaking to providers in person. Conversely, phone interactions seemed to carry an innate pressure to continue the conversation.

*When something was particularly emotional […] it’s easier to take a break when you’re talking to the person than when you’re on the phone. […] You feel like you gotta—you can’t have silences over the phone […] like I felt like was under pressure to keep talking and not have that break (C10).*

Previous literature has found that clients who perceive telehealth as less effective than face-to-face practice often cite communication as their primary reason [36]. While all client participants, except for one, had engaged with telehealth, the preference for face-to-face practice was clear.

Clients from the parenting group program maintained a similar view. Despite recognising the benefits and suitability of telehealth, they discussed the value of connecting face to face.

*I don’t know if [the group] could be a webcam thing […] I think fundamentally the mothers’ groups are about connecting with people and yeah…the online meetings were basically trying to make the best out of an odd situation (C02).*

Limitations of virtual focus groups were noted, with clients wanting to have more personal interactions with peers.

*One of the downsides is that it’s less personal […] so you couldn’t—it’s not like in a group, where you’ve got like seventeen faces there, you can have a personal conversation […] you couldn’t sort of just have your one-on-one time which you might have done in a face-to-face setting (C02).*

These clients also suggested that telehealth limited their ability to dialogue fully with the facilitator and guest speakers. For some, the chat function of the videoconferencing platform made communication more difficult.

*[…] you probably couldn’t, like you couldn’t ask the questions as well as what you wanted to because normally you can just interrupt and ask the questions, but it was a bit—a lot harder when you had to stop and type (C01).*

#### 3.3.4. Client Perceptions and Attitudes towards Telehealth

Clients commonly viewed telehealth as being a temporary substitute for face-to-face services, rather than a distinct mode of practice. While a few clients discussed the benefits and uses of telepractice beyond COVID-19, many regarded it as secondary to traditional in-person services. A recurrent thread across clients’ accounts was that telehealth was a way to ‘*make the best out of a bad situation’*. When asked about whether they would like to see aspects of telehealth integrated into service delivery permanently, many reported they would only use it if they were unable to attend face to face.

*I guess …well, no. Not really, because, you know, the situation we were in was—they were making the best out of a situation […] I guess [it could be used for] all that sort of stuff where obviously people can’t make it or say their kids are sick or they’re sick or something like that (C02).*

In line with some staff attitudes, when given the choice, clients generally felt face-to-face practice would be preferrable. This attitude was most strongly held by those accessing counselling services; in other service areas, views were more varied. Within the parenting support group, there were both those who believed in the primacy of face-to-face practice and those who saw the potential for telehealth to enhance services. Some suggested using telepractice to record information sessions to enable access for women living rurally.

Clients who were engaged with FDR services were, for the most part, neutral towards telehealth. These individuals reported having more experience with telepractice prior to COVID-19 as either they or the person they were mediating with were located outside Geraldton. For these clients, the biggest shift was the increased use of videoconferencing, with previous telehealth being limited to phone. This change was viewed positively, some suggesting that it should be adopted into regular service delivery.

*They should keep Zoom. It works and it’s good and it’s fairly broadly used these days […] the phone works alright. It’s a little bit…Zoom tends to be better in the sense it makes it a little more…a little bit more welcoming, I suppose, or a little bit less clinical (C08).*

## 4. Discussion

This in-depth exploration in a regional setting was undertaken within a single community service agency. It considers the effects of adaptations implemented to enable the continuation of multiple programs during the rapid imposition of changes associated with COVID-19. Qualitative examination of organisational and client experiences of rapidly adapting to telehealth delivery shows that responses to, and perceptions of, telehealth varied substantially amongst both service providers and users. Many themes and subthemes identified are broadly consistent with existing literature. The benefits of telepractice, especially for regional and rural residents [8,12], the challenges associated with telehealth, such as technological barriers [5,8], and concerns around confidentiality, privacy, and safety [37] have been discussed in past studies. The current findings build on previous reports and point to the need for greater consideration of ways in which telepractice can be tailored to the preferences and requirements of different client groups. A discussion of the principal findings, implications for practice and research, and limitations of this study follows.

### 4.1. Principal Findings and Implications for Practice and Future Research

#### 4.1.1. Variation in Experiences

Experiences and perceptions of telehealth varied considerably, with some participants responding positively to the introduction of telepractice and others expressing concerns regarding its effectiveness within the community service space. This was common across staff and client subgroups with an array of attitudes identified, including enthusiasm for telepractice, neutrality, and a strong preference for traditional in-person services. Participants’ experiences seemed, in part, to be shaped by previous experiences with telehealth and levels of comfort and ability using technology. This is congruent with recent research, such as Békés’ and Aafjes-van Doorn’s [38] study of psychotherapists’ engagement with telehealth which found past experiences with online modalities were associated with positive attitudes towards telepractice. In the current study, staff members’ attitudes also appeared to be shaped by their perception of whether telehealth was beneficial to clients, whether they encountered technological challenges, and personal characteristics such as openness to change.

Experiences of telehealth were differentiated between service areas. Those involved in the parenting support group acknowledged both the benefits and challenges of online delivery. Face-to-face services provided clients with a stronger sense of connection, a greater ability to interact with one another and the facilitator, and more value for their child. However, the online platform extended the reach of the program which could enable geographically isolated women to access the service. The current study yielded some suggestions from clients about the structure of online support groups, such as decreasing the duration of sessions to account for the increased tiredness arising from videoconferencing [39] and decreasing the number of participants to allow for greater interaction. The dearth of studies about the use of telehealth to facilitate support groups for new and expecting parents indicates that further research is needed in this area.

Regarding counselling, staff and clients for the most part indicated a preference for face-to-face service delivery, describing it as more effective and valuable for the therapeutic process. Clients from this area emphasised the importance of non-verbal communication, physical presence, and the personal nature of face-to-face services. Clients also suggested that they were more conscious of what they discussed with their counsellor when using telehealth and felt restricted in how open they could be. These results reflect those of Callis et al. [1], who found that over 60% of WA families who accessed mental health services during COVID-19 reported that services met their needs ‘less’ or ‘much less’ than before the pandemic. Callis et al. [1] (p. 20) compared this to other service types, such as food and financial services; 56.7% of WA families accessing food support reported that the services “met their needs just as well or more” than prior to COVID-19. The present study supports this finding; while clients from emergency relief [ER] were unable to be recruited as participants, staff involved in providing food assistance described clients’ responses to telehealth as broadly positive and that clients were more comfortable and open when accessing ER through telehealth.

There are several potential explanations for the notable variation in clients’ experiences between service areas. In the case of ER, previous studies have found that individuals who access these services often report feeling discomfort and embarrassment when seeking support [40]. It is possible that the higher degree of anonymity afforded by telephone appointments offered clients a greater sense of comfort and encouraged self-disclosure. Literature also notes that some groups are more likely to access ER, including those from low-income households, people with disabilities, sole-parent families, and people receiving pension [41]. Individuals from these groups may experience difficulties accessing traditional services due the cost of fuel or transport, mobility issues, or caring responsibilities. Thus, it is possible that the transition to phone appointments was received positively by ER clients as it increased the convenience of accessing the service. Further research exploring the perspectives of ER clients should be undertaken to elicit a clearer understanding of the perceived effectiveness of telehealth in this area.

Previous research has demonstrated an association between “a history of both depression and anxiety” and poorer telehealth experiences [36] (p. 8). This correlation may in part explain the current findings which indicate that clients who accessed mental health support (through the counselling service) perceived telehealth as less effective than in-person visits. The reasons clients were accessing counselling (e.g., lack of informal support systems, experiences of trauma, anxiety) appeared to influence their experiences and perceptions of telehealth. This points to the need for discussions about how providers and organisations can tailor service delivery to the needs of individuals. This echoes Bryant et al.’s [42] (p. 151) assertion that dialogue regarding telehealth must transcend the practicability of technology and consider how ICT can be utilised in a way that is “practice-based” and focused on the end service user.

It is clear from the findings that there is no ‘one size fits all approach’ for using telepractice to deliver community services. Individuals’ experiences varied widely, both within and across service areas. To cater for the multiplicity of needs and circumstances, providers must consider several key factors on a case-by-case basis. Existing literature [43] describes the importance of the following factors: clinical appropriateness, quality (of both the care and technology), safety (regarding informed consent, levels of risk, and security of the telehealth system), and practicalities (including clients’ levels of digital ability and whether clients have the necessary hardware/technology). McClean and Fowler [44] add to this the significance of the service relationship, staff characteristics (confidence, competence, and training), and the characteristics of clients and their circumstances. The current study supports the pertinence of these elements, while also suggesting the importance of an additional factor: clients’ self-assessment of service modalities. Clients articulated which modes of practice were appropriate for their needs, preferences, and specific context. This demonstrates that service users are an important source of knowledge for determining the suitability of telehealth for them and speaks to the need for collaborative processes which view clients as partners in service delivery rather than passive recipients. This study accords with others [45] in asserting that clients’ views and preferences cannot be assumed, and that ongoing review of telehealth is needed to accommodate for service-users’ changing situations and needs.

#### 4.1.2. Centrality of Communication and Relationship

The importance of effective communication and relationships was raised across service areas by both staff and client subgroups. For some, the lack of certain cues, such as body language or facial expressions, greatly impacted their experiences. This was especially true for clients engaged in counselling or other emotional support services. For these respondents, there was a sense that telehealth services were less personal than face-to-face interactions. These results are consistent with Callis et al. [1] (p. 20), who found WA families accessing community services during COVID-19 cited “the loss of face-to-face contact” as significantly negative.

Moreover, the current study found that perceptions of communication and experiences of telehealth appeared to be related; if participants found communication to be effective, they viewed telehealth more positively whereas participants who described communication as more difficult or less personal spoke about telehealth more negatively. This finding is in accord with that of Isautier et al. [36] (p. 6), who found that clients who reported telehealth was worse than in-person services recurrently cited “communication is not as effective as face-to-face visits” as the main reason. This is also consistent with McClean and Fowler’s [44] (p. 12) rapid review of telepractice in human services which found telehealth “can affect the development and maintenance of personal trust and the formation of a therapeutic alliance […] because technology generally limits non-verbal communication”. Therefore, ways of communicating and engaging through telehealth to ensure effective working relationships must be a central consideration.

The current study suggests the importance of relational elements when providing community services through telehealth. It is possible that further training focused specifically on remote engagement and communication could assist practitioners to connect with clients. This supports van Galen et al.’s [46] (p. 714) claim that “telehealth requires expansion of physicians’ communication competencies training”. While van Galen et al.’s [46] work is situated within a medical context, their assertion that telehealth requires different communication competencies and thus additional training appears to resonate with community services context. In particular, the current study points to the need for supporting clients to speak openly, ask questions, and feel comfortable during moments of silence. Building on recommendations made by Sansom-Daly and Bradford [45] and Gordon et al. [47], it appears important to address expectations of telehealth with clients prior to the first session, explicitly naming and exploring some of the above experiences (e.g., “some people find it uncomfortable to have silences over the phone, I’m wondering how you think that would be for you?”). While some providers described using test sessions or ‘dummy runs’ to familiarise clients with the technology, practitioners should also utilise this opportunity to discuss any concerns the client may have about telehealth, explore their individual needs and circumstances, and create a plan for if they encounter technical difficulties during the session.

#### 4.1.3. Issues of Accessibility and Equity

Both clients and staff noted the potential of telepractice to expand the reach of services as well as deliver support in a more flexible and accessible way. The benefits of this for vulnerable groups, such as those living rurally, are well documented (11–12). However, the rapid digitalisation catalysed by COVID-19 has also “highlighted the broad implications of the digital divide” [48] (p. 291). Disparities in digital literacy, access to reliable internet, and ownership of devices or hardware exclude some people from accessing telehealth services [48]. This is of particular concern for regional and rural residents, who tend to fall into lower-income brackets [17] and thus may experience difficulties affording the technology or data needed to support online telehealth sessions [16,49]. While no client participants discussed these issues, such disparities appeared to have impacted on service delivery for providers. Staff described encountering numerous challenges due to clients’ limited internet broadband or poor reception.

The implications of this cannot be understated within the context of community service provision, where clients may be experiencing multiple vulnerabilities preventing them from accessing high-speed internet or the required equipment and facilities to enable their engagement in telehealth and without disruption. The Australian Council of Social Service articulated the significance of this issue in a recent report, suggesting that “digital access and the digital divide have been at the forefront of concerns for the community sector” [49] (p. 18). With anticipations that telehealth will be delivered at higher rates in the foreseeable future [14], it will be critical for community service organisations and providers to carefully consider the digital divide and issues of equity moving forward. This is also an important issue for future research; additional studies exploring clients’ experiences are needed to develop of full picture of how technological equity can be supported within the community service space. In addition, partnering with Consumer Advisory Committees may offer valuable insights about specific community and place-based issues, allowing organisations to examine telehealth through an equity lens and facilitate greater accessibility.

#### 4.1.4. Telepractice beyond COVID-19

Two divergent discourses emerged regarding the future role of telehealth within the community service sector. Among participants, there were some that viewed telepractice as an opportunity to enhance services and increase options for clients and practitioners. Others, however, expressed relief that service delivery could revert to traditional face-to-face modalities, indicating that they would sparsely utilise telehealth in the future. These opposing perspectives are evident in recent literature, with Sanders [5] reporting that regional human service workers were divided when discussing the utility of telehealth. The current study reflects this, with some providers confident that an increased use of telehealth would benefit clients. Staff and clients involved in FDR discussed the value of integrating videoconferencing into service provision beyond COVID-19, conceptualising it as an alternative to phone mediations for those living rurally to offer the added benefit of visuals. It was also seen as a tool for increasing client safety when mediating parties have high levels of conflict.

While some participants were open to integrating telepractice into routine service delivery, others expressed ambivalence, describing it as a modality which is ‘better than nothing’; that is, that telehealth would be a suitable option only when face-to-face practice is unavailable or if extenuating circumstances necessitated it. This view of telehealth has been observed across numerous professions, with Slavova-Azmanova et al. [50] (p. 9) suggesting that there is a “dichotomy of acceptance for telehealth but preference for face-to-face”, which is particularly evident in rural settings. This is shown in the present findings, with some clients and staff expressing a strong preference for in-person services despite their recognition of telehealth’s benefits.

Overall, the current study supports the assertion made by Sansom-Daly and Bradford [45] (p. 1407) that “each modality [telehealth and face-to-face] shines and falters in unique ways”, and that a considered integration of both may be beneficial to cater for the diversity of clients’ needs. To accommodate for the range of experiences and perceptions of telehealth, consumer choice should be prioritised. The current study was undertaken in the context of telehealth being rapidly and ‘forcibly’ implemented; as transition to a state of ‘COVID-normal’ occurs, it will be essential for organisations to re-examine telehealth systems using a client-centred lens. Additionally, to the best of the authors’ knowledge, there are yet to be guidelines developed regarding telepractice in the context of community services. The creation of such a resource may be beneficial for providers in delivering telepractice beyond COVID-19.

### 4.2. Limitations of This Study

The findings of this study should be considered in the context of several limitations. Firstly, the research focused on the experiences of staff and clients from a single community service organisation within regional Australia. While this enabled an in-depth exploration and comparison of multiple service areas, the results may not be transferrable to other populations. In addition, the forms of telehealth under inquiry were primarily videoconferencing and telephone; thus the findings may not be transferrable to other systems of telehealth or uses of ICT.

It should also be noted that most interviews were conducted by telephone due to social distancing measures being in place at the onset of this study. This may have impacted the responses of participants and unintentionally excluded individuals who lacked adequate reception or preferred face-to-face interactions.

Additionally, the current study was not designed to examine how demographic characteristics, such as age, gender, and cultural background, impacted staff and clients’ experiences. While the findings suggest that familiarity with telehealth and some aspects of personality, such as openness to change, may affect perceptions and experiences, further research would be needed to examine demographic factors.

Finally, like most qualitative studies, the numbers interviewed were relatively small and caution should be used when considering the transferability of the results. That said, conceptual depth was achieved, with thick description elicited from individual interviews and the triangulation of client and provider experiences contributing in-depth knowledge to the growing evidence base surrounding telehealth.

## 5. Conclusions

This study explored staff and clients’ experiences of using telehealth in a regional setting to provide and access community services during the COVID-19 pandemic. There was substantial variation in participants’ experiences and perceptions of telehealth. Staff found the rapid digitalisation to be beneficial in some respects, such as increasing the accessibility of services for some clients and providing more options for practice. However, this was accompanied by an acknowledgement of challenges and complexities, such as safety concerns related to family violence and confidentiality, issues of equity for clients who lack ICT or reliable broadband and navigating client hesitance. For both staff and clients, emphasis was placed on the importance of relationships and communication. Clients, for the most part, found telehealth to be accessible; however, they raised issues regarding the suitability of telepractice for their needs and circumstances. Based on these results, several suggestions were made for providers, organisations, and researchers to move towards a greater individualisation of telepractice which caters for clients’ diverse contexts and multiplicity of needs.

## Figures and Tables

**Table 1:** Participant characteristics: Clients and staff.

Characteristics		Clients (11) *n* (%)	Staff (15) *n* (%)
Service Areas	Counselling	5 (45)	6 (40)
	Family separation service	3 (27)	5 (33)
	Family and parenting support services	3 (27)	1 (7)
	Emergency relief	0 (0)	1 (7)
	More than one service area	0 (0)	2 (13)
Gender	Male	2 (18)	1 (7)
	Female	9 (82)	14 (93)
Age (years) ^1^	25–34	2 (18)	3 (20)
	35–44	5 (45)	3 (20)
	45–54	1 (9)	3 (20)
	55–64	0 (0)	3 (20)
	65–74	1 (9)	3 (20)

^1^ The ages of two client participants have been excluded due to imprecision (e.g., response of ‘over 40′).

## Data Availability

Data sharing not applicable.
